# Supplementary material for: A sepsis treatment algorithm to improve early antibiotic de-escalation while maintaining adequacy of coverage (Early-IDEAS): A prospective observational study
Source: PLoS One. 2023 Dec 20;18(12):e0295908. doi: 10.1371/journal.pone.0295908 (PMC10732396; doi:10.1371/journal.pone.0295908)
Supplement: Attachment — Submitted filename: Response to reviewers 2452023.docx [file pone.0295908.s001.docx]

May 24^th^, 2023

Rose Ann Joyce Sagun Puetes

Academic Editor

PLOS ONE

RE: A sepsis treatment algorithm to improve early antibiotic de-escalation while maintaining adequacy of coverage (Early-IDEAS): A prospective observational study (PONE-D-23-03428)

Dear Dr. Puetes,

Thank you for your consideration of our manuscript **"A sepsis treatment algorithm to improve early antibiotic de-escalation while maintaining adequacy of coverage (Early-IDEAS): A prospective observational study”**, for publication in PLOS ONE. We just wanted to bring to your attention that the following remark:” *In the Methods section please revise the informed consent statement to reflect whether written/verbal informed consent was obtained from all participants for inclusion in the study*”, has been addressed as per the comments received from Dr. Paula Katrina A. Maderazo on April 29th, 2023. Our submission on May 8th, in **(L183-185, L188)** on the “Revised manuscript with track changes” document, stated “*The ethics committee of Sunnybrook Research Institute has waived ethics approval and individual consent requirement for this project as it was deemed a quality improvement project by Sunnybrook Research Institute Ethics Review – Self Assessment Tool (ER–SAT).*”

We look forward to hearing from you.

Sincerely,

Mohamed AGA Bucheeri, MB BCh BAO

Medical Resident

Postgraduate Medical Education, University of Toronto

Toronto, Canada

Derek R MacFadden MD ScD

Clinician Scientist

The Ottawa Hospital Research Institute

Ottawa, Canada

April 19^th^, 2023

Herman Tse

Academic Editor

PLOS ONE

RE: A sepsis treatment algorithm to improve early antibiotic de-escalation while maintaining adequacy of coverage (Early-IDEAS): A prospective observational study (PONE-D-23-03428)

Dear Dr. Tse,

Thank you for your consideration of our manuscript **"A sepsis treatment algorithm to improve early antibiotic de-escalation while maintaining adequacy of coverage (Early-IDEAS): A prospective observational study”**, for publication in PLOS ONE. We greatly appreciated the thoughtful comments and have strived to incorporate the suggestions into the manuscript. We believe the paper has been strengthened in the process. The detailed point-by-point responses to the comments are provided below. We have included both ‘tracked changes’ and ‘unmarked’ versions of the updated manuscript with the revision. Please note that the line numbers below correspond to the “Revised manuscript with track changes” document.

We look forward to hearing from you.

Sincerely,

Mohamed AGA Bucheeri, MB BCh BAO

Medical Resident

Postgraduate Medical Education, University of Toronto

Toronto, Canada

Derek R MacFadden MD ScD

Clinician Scientist

The Ottawa Hospital Research Institute

Ottawa, Canada

***The present decision is based on my inspection of the manuscript without input from external peer reviewers yet. This is because there are several major issues with the results which require clarification before the manuscript could be sent out for review. These are:***

***1. Table 2 - the current presentation is confusing; the text (L253-261) accompanying the table described the recommendations in terms of patients, but the table presented the recommendations in relation to each antibiotic.***

***Furthermore, it's unclear how the table is divided into 2 halves, antibiotics prescribed by team and recommended by algorithm. For example, for "no change" in ceftriaxone and meropenem, the numbers are 20 and 1 in the first half, but 22 and 2 in the second half.***

The text gives a brief overview of antibiotics that were either given (prescribed by the team) or recommended (by the algorithm). Each of these two arms is further subdivided by whether they were to be de-escalated by the algorithm, escalated, or not changed (‘no change’). This allows the reader to compare what antibiotics were being given clinically versus what they would have been changed to according to the algorithm. Based on this feedback, we have made changes to the text **(L315-339)** and Table 2 to make this more interpretable. We feel these comparisons are important to the reader, and with these new changes, we feel they are now much easier to understand/interpret.

We have now included clarification of what circumstances were defined as ‘No change’ in the methods section (**L249-260**) and is as follows: “If antibiotics were not changed, then these were considered as ‘No change’ in spectrum. In some instances, antibiotics were changed to those not included in our antibiotic cascade (e.g., levofloxacin for community acquired pneumonia by local guidelines), or atypical antimicrobial coverage was added (e.g., azithromycin for atypical organism coverage in CAP). For these instances we typically considered these guideline-based changes as equivalent in spectrum (e.g., we classified guideline directed prescribing with levofloxacin or ceftriaxone/azithromycin for CAP as equivalent spectrum-level). We also assumed no change in spectrum-level with the addition of atypical or anaerobic specific adjunctive agents such as azithromycin or metronidazole. We considered the following agents not listed in our antibiotic cascade as equivalent levels of spectrum (i.e., there would be no change in spectrum with a change of agent): (1) Ertapenem and Meropenem; and (2) ceftazidime and gentamicin.”

We did identify one discrepancy in summation of counts in Table 2 and this was corrected, without significant changes in our findings.

We have also moved the summaries of antibiotic escalation/de-escalation to a new Table 3, which allows us to present these in a more interpretable fashion.

***2. Table 3 - similar issue as table 2. It's unclear how the numbers for each recommendation category should be interpreted with respect to each column. Also, for this patient subgroup (n=37), it can be inferred that 5 have both positive blood and non-sterile site cultures positive results, but the antibiotics given would obviously be systemic and apply to both (in most cases, though there are exceptions like daptomycin being useful for bacteremia but not pneumonia). It would be important to verify that the antibiotics given or recommended be adequate to cover both categories of positive cultures.***

We have decided to remove this Table 3, as it is challenging to interpret, and instead provide simple summary descriptions in the text. As a result, **(L387-393, L498-506)** have been updated accordingly.

We have also added to this text description **(L501-506)** the results for the smaller number of patients that had both blood and non-blood cultures positive.

Lastly, we have added text **(L579-581)** emphasizing the fact that the antibiotics evaluated in this study (and recommended by the Early-IDEAS algorithm) were systemic antibiotics with distribution to blood and penetration to most non-sterile sites**.**

***3. While keeping in mind that this is a pilot study, there are some notable results from table 3, (a) no difference in treatment adequacy between clinical team's prescription and algorithmic recommendations, and (b) there were more escalations than "no change" and de-escalations combined. When considered together, it appears reasonable to infer that the recommended escalations were unnecessary and/or inappropriate. Of course, this might be a misinterpretation due to issues with data presentation as mentioned above. Nonetheless, a more analytical discussion of the results in this subgroup would be necessary in the revision.***

We agree that these are rather misinterpretations of a somewhat challenging tabular representation of data. We think the changes we have made (Clarifying and splitting Table 2 into Table 2 and Table 3), along with the removal of the previous Table 3, will better highlight our findings. Namely, the algorithm can achieve antibiotic de-escalation across a large proportion of patients while maintaining comparable (and, in fact, identical) empiric antibiotic treatment adequacy proportions.
